# Mitochondrial Health Index Correlates with Plasma Circulating Cell-Free Mitochondrial DNA in Bipolar Disorder

**DOI:** 10.21203/rs.3.rs-2821492/v1

**Published:** 2023-04-26

**Authors:** Giselli Scaini, Rafaela Cordeiro, Camila Carvalho Lima, Gabriel Fries, Giovana Zunta-Soares, Jair C. Soares, Joao de Quevedo

**Affiliations:** Faillace Department of Psychiatry and Behavioral Sciences; Faillace Department of Psychiatry and Behavioral Sciences; Faillace Department of Psychiatry and Behavioral Sciences; University of Texas Health Science Center at Houston; The University of Texas Health Science Center at Houston; The University of Texas Health Science Center at Houston

**Keywords:** Bipolar Disorder, Depression, Mitochondria, Mitochondrial Health Index, Mitochondrial Quality Control, ccf-mtDNA

## Abstract

**Background:**

Although mitochondria dysfunction is known to play an essential role in the pathophysiology of bipolar disorder (BD), there is a glaring gap in our understanding of how mitochondrial dysfunction can modulate clinical phenotypes. This study aimed to evaluate the composite mitochondrial health index (MHI) in BD subjects and non-psychiatry controls (Non-psychiatry controls). We will also explore whether lower MIH will be related to higher cell-free mtDNA (ccf-mtDNA) levels and poor clinical outcomes.

**Methods:**

Fourteen BD-I patients and 16 age- and sex-matched non-psychiatry controls were enrolled for this study. Peripheral blood mononuclear cells (PBMCs) were used to measure the enzymatic activities of citrate synthase and complexes I, II, and IV and mtDNA copy number. ccf-mtDNA was evaluated by qPCR in plasma. Mitochondrial quality control (MQC) proteins were evaluated by western blotting.

**Results:**

One-Way ANCOVA after controlling for age, sex, body mass index (BMI), and smoking status showed that patients with BD present a decrease in the MHI compared to non-psychiatry controls, and higher ccf-mtDNA levels, which was negatively correlated with MHI. Because the MQC network is essential to maintain mitochondrial health, we also evaluated the relationship between MQC-related proteins with MHI and ccf-mtDNA. Our results showed that MHI negatively correlated with Fis-1 and positively with Opa-1 and LC3. Moreover, we found a negative correlation between ccf-mtDNA, Opa-1, and LC3 and a positive correlation between cff-mtDNA and Fis-1. Finally, we found that subjects with longer illness duration, higher depressive symptom scores, and worse functional status had lower MHI and higher ccf-mtDNA.

**Conclusion:**

In summary, the present findings corroborate previous studies and provide strong support for the hypothesis that mitochondrial regulation and function are integral parts of the pathogenesis of BD.

## Introduction

Bipolar disorder (BD) is a severe and chronic psychiatric disorder that affects approximately 1–4% of the world population ^1^. BD seems to be a steadily growing threat to the health systems since current treatment options remain unsatisfactory, and many patients continue to experience intra-episodic symptoms or even full-blown episodes resistant to treatment ^2^. The pathophysiological pathways responsible for BD remain elusive even after many years of research, likely due to multifactorial etiology involving the interaction between multiple genetic, neurochemical, and environmental factors ^3^.

The mitochondrial dysfunction hypothesis has been corroborated by several studies showing that BD patients present an atypical mitochondrial metabolism, oxidative stress, abnormal mitochondrial morphology and dynamics, and mitochondrial DNA (mtDNA) damage ^4^. Genetic studies also supported this hypothesis, showing an increased likelihood of maternal inheritance in generational transmission of BD, abnormal findings in the mtDNA of patients with BD, and comorbidity of affective disorder with mitochondrial diseases ^5–7^. Moreover, chronic stress (e.g., recurrent affective episodes) can lead to an accumulation of mitochondrial dysfunction – or mitochondrial allostatic load – which might represent an early event that increases allostatic load and disease risk in BD ^8, 9^.

To disentangle these contributors and identify the molecular nature of a potential mitochondrial perturbation, a mitochondrial health index (MHI) was developed to assess mitochondrial functional capacity in human leukocytes ^10^. This metric integrates nuclear and mitochondrial DNA-encoded respiratory chain enzymatic activities and mtDNA copy number (mtDNAcn) into an index reflecting mitochondrial respiratory chain (RC) capacity per-mitochondrion basis. The MHI was previously found to be low among highly stressed caregivers compared to controls, and the MHI was also associated with mood states in this group ^10^. While these findings are novel and interesting, the MHI has not, to date, been applied to BD, and its clinical relevance remains unknown.

Although preliminary studies from our group suggest that BD patients present an impairment in mitochondrial dynamics and mitophagy, it is unknown whether the failure to clear damaged mitochondria mediates the decrease in mitochondrial function. We hypothesize that BD patients will show lower MHI due to changes in the MQC network, which will be associated with higher cell-free mtDNA (ccf-mtDNA) levels and poor clinical outcomes. To test this hypothesis, in this preliminary study, we evaluate the activities of mitochondrial complexes I, II, IV, citrate synthase (CS), and mtDNAcn, to further calculate the composite MHI in peripheral blood mononuclear cells (PBMCs) from patients with BD and non-psychiatric controls. We also evaluate the levels of proteins involved in mitochondrial dynamics, mitophagy, active caspase-3, and ccf-mtDNA levels.

## Subjects And Methods

### Subjects

This study was carried out in accordance with the principles of the Declaration of Helsinki with approval from the Institutional Review Board of the University of Texas Health Science Center at Houston (HSC-MS-09-0340), and written informed consent was obtained from all research participants. Fourteen participants with BD type I (BD-I) were recruited from the UTHealth Mood Disorders outpatient clinic and sixteen non-psychiatry controls were recruited from the local community who did not have a personal psychiatric disorder or family history of major psychiatry disorder or neurologic disorders in first-degree relatives. Patients and non-psychiatry controls were matched for ethnicity, age, and sex. Patients and non-psychiatry controls were assessed with the Mini-International Neuropsychiatric Interview (MINI) to confirm BD diagnosis (patients) or to exclude a history of psychiatric disorders (controls) ^11^. BD participants were also assessed using the Montgomery-Åsberg Depression Rating Scale (MADRS) ^12^ and the Young Mania Rating Scale (YMRS) ^13^ to index the severity of depressive and manic symptoms, respectively. Functioning was assessed with the Global Assessment of Functioning (GAF) Scale and Functioning Assessment Short Test (FAST) ^14, 15^.

### Processing Whole Blood Samples

Human blood samples were collected in heparin-coated collection tubes. Then, PBMCs were separated using LeucoPREP brand cell separation tubes (Becton Dickinson Labware, Lincoln Park, NJ, USA). PBMC cell pellets were mixed with RPMI-1640 medium containing 10% DMSO and frozen overnight in a Mr. Frosty container with 2-propanol (#5100–0001, Nalgene, Rochester, NY) at − 80°C following an appropriate post-processing delay. Preanalytical characterization and quality control (QC) were performed using trypan blue-based methods to evaluate cell viability after (post-thaw) cryopreservation. Our results showed that cell viability remained above 70% in all samples.

### Mitochondrial Enzymatic Activities

PBMC pellets were mechanically homogenized in a homogenization buffer extraction buffer containing 1mM EDTA and 50mM triethanolamine to release individual enzymes. Then the homogenate was used to quantify the enzymatic activities of enzymatic activities citrate synthase, complex I, complex II, and cytochrome c oxidase (complex IV) using kinetic spectrophotometric assays. On the day of the assays, the samples were frozen and thawed in hypotonic assay buffer three times to expose the enzymes to substrates and achieve maximal activities fully. Citrate synthase activity was assayed according to the method described by Srere ^16^, measuring the formation of the -SH group released from CoA-SH using the reactive Ellman reagent (5,5`-dithiobis [2-nitrobenzoic], DTNB) and monitoring the absorbance at 412 nm. NADH dehydrogenase (complex I) was evaluated according to the method described by Cassina and Radi ^17^ by the rate of NADH-dependent ferricyanide reduction at 420 nm. The activity of succinate: 2,6-dichlorophenolindophenol (DCIP) oxidoreductase (complex II) was determined according to the method of Fischer and colleagues ^18^. Complex II activity was measured following the decrease in absorbance due to the reduction of 2,6-DCIP at 600 nm. The activity of cytochrome c oxidase (complex IV) was assayed according to the method described by Rustin and colleagues ^19^, measured by following the decrease in absorbance due to the oxidation of previously reduced cytochrome c at 550 nm. The specific activity for each enzyme was obtained by calculating the slope (first derivative) of the optical density change and subtracting non-specific activity detected in the presence of specific inhibitors for each complex or in the absence of the rate-limiting reaction substrate. All assays were performed in triplicates at 30°C, and final values were normalized on a per-cell basis using the qPCR-based estimates of cell numbers for each biological sample as described in Picard et al ^10^.

### Mtdna Copy Number

Real-time quantitative PCRs were performed to measure the amount of mtDNA relative to a single-copy gene (beta-hemoglobin) with a modified protocol from Tyrka et al ^20^. Reactions included 25 ng genomic DNA, 300 nmol l − 1 of each primer, and 1 × Sybr Select Master Mix (Life Technologies, Carlsbad, CA, USA) in a final volume of 10 µl. Primer sequences and PCR cycling conditions for both mtDNA and beta-hemoglobin have been previously reported ^20^. Reactions were carried out in 96-well plates, and data were acquired in a QuantStudio 7 Flex Real-Time PCR System (Life Technologies). mtDNAcn for each sample was determined by relative quantification based on a 5-point standard curve performed with a serial dilution (1:2) of a calibrator sample ranging from 1 to 0.0625 ng DNA. All samples were analyzed in triplicate. The relative amount of mtDNA was finally divided by the relative amount of the beta-hemoglobin gene to obtain an index of mtDNAcn.

### Rationale For Calculating The Mhi

The MHI was computed by integrating three enzymatic measures of respiratory chain capacity and two mitochondrial content features, as described previously ^10, 21^. To calculate the MHI, the four mitochondrial features were mean-centered, so each parameter would contribute an equal weight in the equation. Combining three features (complexes I, II, and IV) as a numerator, divided by two content features (CS and mtDNAcn) as a denominator produces a quantitative index of mitochondrial energy production capacity, or “quality” on a per cell mitochondrion basis, where a value of 100 represents the average of the cohort, and values > 100 and < 100, respectively, indicate higher and lower respiratory chain capacity on a per mitochondrion basis. Previously, the composite MHI exhibited a higher predictive ability of caregiver (chronic stress) status than any of the individual MHI components alone ^10, 21^.

### Immunoblotting

To perform the immunoblotting experiments, isolated PBMCs were lysed in 100 µL of lysis buffer (60 mM Tris-HCl [pH 6.8], 2% SDS, and 10% sucrose) supplemented with 1:100 protease inhibitor cocktail (Sigma-Aldrich, St. Louis, MO, USA), followed by sonication and determination of protein concentration using a BCA kit (Thermo Fisher Scientific, 23252). Equal amounts of proteins (30 µg/well) were fractionated using a 4–20% precast polyacrylamide gel (Bio-Rad) and electroblotted onto a PVDF membrane using the Trans-Blot Turbo transfer system (Bio-Rad, Hercules, California, USA). Afterward, the membranes were blocked in Tween-Tris-buffered saline (TTBS: 100 mM Tris-HCl [pH 7.5] containing 0.9% NaCl and 0.1% Tween-20) containing 5% nonfat dry milk. The membranes were incubated overnight at 4°C with primary antibodies against Mfn-2 (Abcam, ab56889), Opa-1 (Invitrogen, MA5-16149), Fis-1 (Abcam, ab156865), Parkin (Santa Cruz Biotechnology, Inc., sc32282), p62/SQSTM1 (Cell Signaling Technology Inc., #8025), and LC3 (Abcam, ab52628). Then, the primary antibodies were removed, and the membranes were washed 4 times for 15 min. After washing, an anti-rabbit or anti-mouse IgG peroxidase-linked secondary antibody was incubated with the membranes for 1 h (1:5,000 dilution), and the membranes were rewashed. Finally, immunoreactivity was detected using an enhanced chemiluminescence ECL Plus kit (Bio-Rad, Hercules, California, USA). After exposure, the membranes were stripped and incubated with a mouse monoclonal antibody against β-actin (Sigma-Aldrich, A2228). An anti-mouse IgG peroxidase-linked secondary antibody was incubated with the membranes for 1 h (1:10,000 dilution), and the membranes were rewashed. The signals were visualized using an ECL detection reagent (Bio-Rad) and monitored using a ChemiDoc MP imaging system (Bio-Rad, Hercules, California, USA).

### Plasma Circulating Cell-free Mitochondrial Dna (Ccf-mtdna)

DNA was isolated from thawed plasma samples from the same subjects included in the study using the QIAmp 96 DNA Blood Kit (Qiagen, Valencia, CA, USA), according to the manufacturer’s instructions. The quantitative analysis of ccf-mtDNA was performed using real-time PCR using SYBR Green Technology (Thermo Fisher Scientific, Waltham, MA, USA) according to Pyle et al ^22^. The different crossing-point values from the unknown samples were compared with the standard curve, and the corresponding number of mitochondrial units were calculated. The amount of DNA (g µl^− 1^) was divided by the size of the PCR fragment (bp) and the molar mass per base pair (g mol^− 1^).

### Statistical analysis

Statistical analyses were performed using Statistical Package for the Social Sciences, v.23.0 (SPSS Inc., USA). The normality of data distribution was assessed using the Shapiro-Wilk test and histogram visualization. The subjects’ demographic and clinical characteristics were presented in tables as percentages, mean (SD), or median (interquartile range), according to distribution data. Chi-squared was applied for statistical comparisons between the categorical variables. Age, body mass index (BMI), MADRS, YMRS, FAST, and GAF showed nonparametric distributions and were therefore analyzed by the Mann-Whitney U test.

To ensure that linear regression models met their assumptions, regression diagnostics were performed. Shapiro-Wilk tests were used to determine whether the data distributions were normal. For variables that did not follow a normal distribution, natural logarithms were applied to ensure normality. In addition, standardized residual plots versus standardized predicted values were used to detect linearity and homoscedasticity. Each model was tested for multicollinearity using tolerance criteria and variance inflation factors (VIFs). Diagnostic tests were passed by all models.

Analysis of covariance (ANCOVA) was performed to determine the relationship between groups with the addition of age, sex, BMI, and self-report smoking status (yes/no) to check for significant effects and interactions since these factors are thought to interfere with mitochondrial function ^23–25^. If an interaction was significant, Bonferroni corrected post-hoc test was performed (P < 0.05, two-tailed). Correlations between variables were tested with either Pearson’s or Spearman’s tests depending on their distribution. All statistical tests were two-tailed and p values < 0.05 were considered statistically significant after Bonferroni correction for multiple testing. Linear regression was performed with MADRS, YMRS, GFA, FAST, MHI and ccf-mtDNA as the dependent variables, and MHI, ccf-mtDNA, length of illness as predictor, controlling for sex, age, BMI and self-report smoking status (yes/no).

## Results

Characteristics of BD participants and non-psychiatry controls are shown in Table 1. Notably, no significant differences in sociodemographic factors (ethnicity, age, sex, BMI, and smoking status) emerged between groups. The mean YMRS score of the BD group was 7.93 ± 7.51, and their mean MADRS score was 17.43 ± 10.61. Moreover, a significant difference in functional status, assessed by GAF (p < 0.001) and FAST (p < 0.001), was found between non-psychiatry controls and patients with BD. All patients were on treatment with various psychiatric medications at conventional doses at the time of the study, including antipsychotics, anticonvulsants, mood stabilizers, and antidepressants. Some patients received additional benzodiazepines and stimulants.

**Figure 1:**
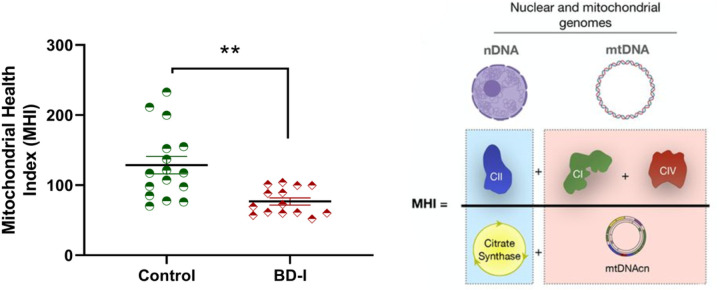
Mitochondrial Health Index (MHI) [mathematical integration of two nuclear DNA (nDNA)-encoded components (left), and mtDNA-related components (right)] in peripheral blood mononuclear cells (PBMCs) from non-psychiatry controls (control) and patients with Bipolar Disorder (BD). Data are presented as mean ± standard error of the mean and were analyzed with univariate generalized linear models with adjustment for sex, age, BMI, and smoking status. ** Different from the control group, p < 0.001.

One-Way ANCOVA, after controlling for age, sex, BMI, and smoking status, showed that patients with BD present lower enzymatic activity of the citrate synthase and complexes I, II, and IV when compared to Non-psychiatry controls. However, the mtDNAcn yielded no statistically significant group differences (Table 2). The present study is the first to demonstrate that patients with BD had a lower MHI than non-psychiatry controls after adjusting for confounding variables (Fig. 1).

We have previously identified an impairment of the MQC in BD patients, described by an imbalance in mitochondrial dynamics towards fission and reduced levels of proteins responsible for removing damaged mitochondria via mitophagy, followed by apoptosis activation ^26, 27^. In the present study, as previously shown by our group, BD patients presented lower levels of Mfn-2, Opa-1, Parkin, p62/SQSTM1, and LC3, while the levels of Fis-1 and active caspase-3 were higher when compared to non-psychiatry controls (Sup. Table 1). Because the MQC system is essential to maintain mitochondrial health, we evaluated the relationship between MQC-related proteins and MHI. Our results showed that subjects with higher levels of Fis-1 exhibited lower MHI (rho = −0.579, p = 0.001), while subjects with higher levels of Opa-1 (rho = 0.616, p < 0.001) and LC3 (rho = 0.600, p = 0.001) had higher MHI (Sup. Table 2).

**Figure 2:**
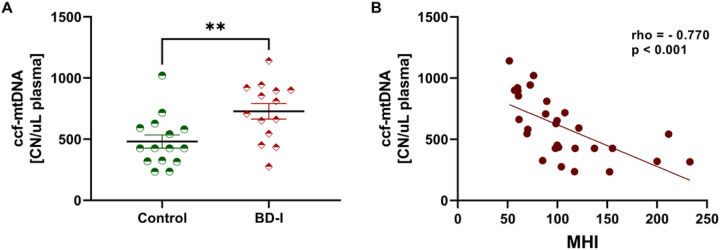
Plasma circulating cell-free mitochondrial DNA (ccf-mtDNA) (A) and correlation coefficient between ccf-mtDNA and mitochondrial health index (MHI) (B) in non-psychiatry controls (control) and patients with Bipolar Disorder (BD). (A) Data are presented as mean ± standard error of the mean and were analyzed with univariate generalized linear models with adjustment for sex, age, BMI, and smoking status. ** Different from the control group, p < 0.001. (B) Results were assessed using Spearman’s correlation test.

Since an imbalance between the MQC network and apoptosis activation can lead to the release of mtDNA into the circulation, we evaluated ccf-mtDNA and whether MQC-related proteins, active caspase-3, and MHI could be associated with ccf-mtDNA. Our preliminary data showed that BD patients had higher levels of ccf-mtDNA (Fig. 2A), which was negatively correlated with MHI (Fig. 2B). In order to investigate whether MHI predicted ccf-mtDNA, we performed linear logistic regression models including age, sex, BMI, and smoking status as covariates. Linear regression analysis showed a negative significant predictive relationship between MHI and ccf-mtDNA after controlling for age, sex, BMI and smoking status (R^2^adj = 0.319, F(5, 23) = 3.629, p = 0.014, β = −3.601 p < 0.001). Moreover, our preliminary analyses showed that subjects with higher levels of ccf-mtDNA had higher levels of Fis-1 (rho = 0.538, p = 0.003) and lower levels of Opa-1 (rho = −0.441, p = 0.017) and LC3 (rho = −0.501, p = 0.008) (Sup. Table 2). Additionally, we found that patients with higher levels of active caspase-3 had lower MHI (rho = −0.512, p = 0.005) and higher ccf-mtDNA levels (rho = 0.637, p < 0.001) (Sup. Table 2).

**Figure 3:**
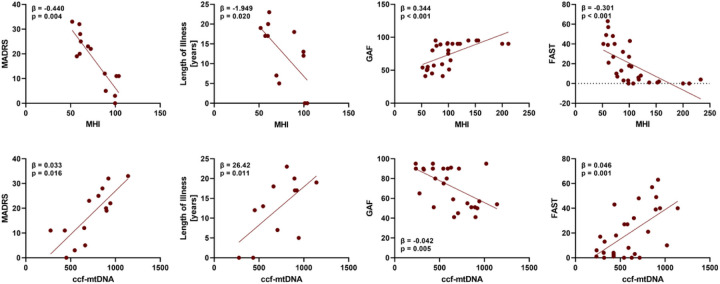
Scatter plot for association between mitochondrial health index (MHI) and circulating cell-free mitochondrial DNA (ccf-mtDNA) with Montgomery-Asberg Depression Scale (MADRS), Length of Illness, Global Assessment of Functioning (GAF) and Functional Assessment Screening Tool (FAST) scale.

In order to investigate whether MHI and ccf-mtDNA levels predicted mood symptoms and functional impairment we performed linear regression models relating MHI and ccf-mtDNA to one measure of mood (MADRS and YMRS), functional status (GAF and FAST), or clinical status (length of illness), controlling for age, sex, BMI and self-reported smoking status. As can be seen in Table 3, within individuals with BD, MHI and ccf-mtDNA predicted the severity of depressive symptoms, while no association was found with YMRS score. Moreover, we observed a significantly negative predictive relationship between length of illness and MHI, and a positive predictive relationship between length of illness and ccf-mtDNA, after controlling for covariates listed above, suggesting that length of illness may be partly responsible for the lower MHI and higher ccf-mtDNA levels observed in BD patients (Table 3). A similar set of analyses, controlling for age, sex, BMI and smoking status, was then performed in the combined BD and non-psychiatry controls samples, to explore the association of MHI and ccf-mtDNA with functional status. Our results showed that participants with lower MHI and higher ccf-mtDNA levels had worse functional status, highlighting that altered mitochondrial functioning can be significant functional deficits (Table 3). Associations from selected models are highlighted in Fig. 3.

## Discussion

Recent studies suggest a non-energetic role of mitochondrial as a new paradigm where mitochondria play a role in the adaptation to stress, having an impact on cellular resilience and acting as a source of systemic allostatic load, when the mitochondria lose the ability to recalibrate and maintain cell omeostasis through changes to mitochondrial morphology, dynamics, and function, a process known as mitochondrial allostatic load ^8, 28, 29^.

Although our preliminary studies found that BD patients have an impairment in the MQC, the downstream effects of these alterations on the MHI and ccf-mtDNA levels are unknown. To the best of our knowledge, this is the first study to investigate the MHI in patients with BD and the association of a decrease in the MHI with alterations in the MQC system, ccf-mtDNA levels, and clinical outcomes. This preliminary cross-sectional study indicates that BD patients have a decrease in mitochondrial enzymes and MHI, reflecting lower mitochondrial bioenergetic capacity, which could result in increased oxidative stress, decreased mitochondrial Ca^2+^ buffering capacity, and loss of ATP, all of which are factors that were described in BD ^4, 30^. Studies have shown that alterations in proteins involved in mitochondrial fusion and fission lead to altered mitochondrial shape, loss of mtDNA, decreased mitochondrial respiration, increased oxidative stress, and apoptotic cell death ^31, 32^. Our first hypothesis was that subjects with lower MHI would present alterations in the protein levels of MQC. Indeed, our data showed that subjects (BD and Non-psychiatry controls) with an increase in the levels of Fis-1 [a protein involved in mitochondrial fragmentation] present lower MHI. On the other hand, subjects (BD and Non-psychiatry controls) with higher MHI showed higher levels of Opa-1 [a protein involved in the maintenance of the respiratory chain and membrane potential, cristae organization, and control of apoptosis] and LC3 [a key component of autophagy/mitophagy], suggesting that mitochondrial fusion may account for a higher bioenergetic capacity. In contrast, mitochondrial fission is associated with a decrease in the bioenergetic efficiency of the cells.

As described above, maintaining a healthy mitochondrial pool is critically regulated by MQC, and inhibiting the mitophagy pathway could lead to a pronounced accumulation of damaged mitochondria and apoptosis ^33^. Moreover, studies have suggested that during the apoptotic process, fragments of mtDNA are released to the extracellular space and may behave as damage-associated molecular patterns (DAMPs) ^34, 35^, leading to a chronic inflammation without microbial infection – termed sterile inflammation – by the activation of various pro-inflammatory signaling pathways ^36–39^. Thus, our second hypothesis was that higher ccf-mtDNA levels would be associated with lower MHI, MQC impairment, and apoptosis activation. Supporting this hypothesis, our preliminary findings demonstrated that patients with BD had higher plasma levels of ccf-mtDNA, which were negatively correlated with Opa-1 and LC3. Moreover, we found a positive correlation between ccf-mtDNA and Fis-1. Finally, we observed that subjects with lower MHI and higher ccf-mtDNA also had higher active caspase-3 levels. Taken together, our results may indicate that BD patients have an increase in mitochondrial fragmentation associated with a decrease in the mitochondrial bioenergetics’ capacity, followed by a lack of mitophagy activation, which could lead to accumulation of damaged mitochondria in the cells, shifting the cell signaling to apoptosis activation. Consequently, fragments of the mtDNA are subsequently released into the circulation and trigger inflammation, a consistent feature of BD’s pathophysiology ^40^.

Increasing evidence indicates that BD is a systemic disorder that affects not only the brain but also peripheral systems, leading to systemic comorbidities that may represent the result of cumulative “wear and tear” from long-term exposure to stress ^41–43^. As a result, these comorbidities can influence and further deteriorate brain function, contributing to accelerated aging and neuroprogression ^44–46^. In this context, in an exploratory analysis, we investigated the correlation of MHI and ccf-mt-DNA with depressive and manic symptoms severity and functional status. Our results showed that depressive and manic symptoms were associated with lower mitochondrial content and bioenergetic capacity and increased levels of ccf-mtDNA. Our study also found an interesting correlation between worse functional status (as shown by lower GAF and higher FAST scores) with lower MHI and higher ccf-mtDNA levels. Poor functioning is considered a key factor of disability in patients with BD-l ^47^. Furthermore, the length of illness has been found to predict MHI and ccf-mtDNA in BD patients, with every 1-unit increase in length of illness resulting in a decreased MHI of 1.949 and increased ccf-mtDNA of 26.42, after controlling for age, sex, and smoking status. Using a machine learning approach, Sartori et al ^48^ demonstrated that brain volume changes in MRI were predictors of FAST scores in patients with BD and could identify specific brain areas related to functioning impairment. Notably, studies have shown that a substantial proportion of patients with BD experience unfavorable functioning in community and outpatient samples, suggesting a significant degree of morbidity and dysfunction associated with BD, even during remission periods ^49, 50^. The FAST score was also reported as sensitive to detecting functioning impairment and accurate in distinguishing early from late stages of BD ^51^.

Regardless of whether mitochondrial dysfunction plays a primary or secondary role, our findings, which align with the literature, indicate that patients with BD present multifactorial alterations in mitochondrial biology. Together, these alterations can lead to mitochondrial allostatic load, which, in turn, contributes to disease progression and poor outcomes via multiple mechanisms, such as gene expression and epigenomics, changes in brain structure and function, abnormal stress reactions, inflammation, and cellular aging. Moreover, because mitochondria are found in all organs, mitochondrial allostatic load affects multiple types of cells and organ systems, resulting in the coexistence of various diseases and symptoms.

There were several limitations in our study. First, we cannot rule out the possibility of a type I error in this preliminary study since it is a cross-sectional study with small sample size. Second, it is known that a single analysis of study participants’ peripheral blood is not the most appropriate strategy as directionality and causality cannot be established. Third, the present study did not account for several factors that might impact mitochondrial health dynamically, including lifestyle factors, childhood trauma, chronic stress, suicidal behavior, acute mood states, exercise, and dietary habits. Fourth, the use of PBMC cell mixtures, rather than specific purified cell types, could have reduced our ability to detect mitochondrial alterations with greater sensitivity and specificity among particular cell populations ^21^. Fifth, most patients were taking one or more psychotropic medications, introducing the confounding effect of polypharmacy. Last, despite uncovering an association between MHI and ccf-mtDNA levels with several clinical variables indirectly linked to clinical severity and progression, another limitation of our study includes the absence of information on the number of previous episodes and hospitalizations. Therefore, our results should be seen as exploratory and require replication and validation. Future research efforts to evaluate mitochondrial functions and related aspects of cellular bioenergetics should be investigated with the proper methodology, using a large sample size, prospective and longitudinal study designs, including repeated measures from patients with BD during all affective states, and statistical adjustment for a range of relevant demographical and lifestyle variables are warranted.

While our findings need replication, they indicate a role for mitochondrial allostatic load in BD, suggesting that in patients with BD, mitochondria represent a potential biological intersection point that could contribute to impaired cellular resilience, increasing the vulnerability to stress and mood episodes, potentially linking mitochondrial dysfunction with the progression of the disease and poor outcomes.

## Figures and Tables

**Table 1:** Demographic characteristics of all subjects

	Healthy Subjects	Bipolar Subjects	p-Value
n	16	14	
Mean age, years	33.75 ± 8.85	35.85 ± 9.00	0.334^a^
Female Gender, n (%)	8 (50%)	8 (57.1%)	0.696^b^
Race/Ethnicity, n (%)			
White/Caucasian	14 (87.5%)	10 (71.4%)	0.272^b^
Hispanic/Latinx	6 (37.5%)	8 (57.2%)	
African American	9 (56.3%)	5 (35.7%)	
Asian	1 (6.3%)	1 (71%)	
Body mass index, mean ± SD	27.46 ± 5.83	27.90 ± 6.29	0.728^a^
Smoking, %	1.0%	28.6%	0.102^a^
MADRS, mean ± SD	0.19 ± 0.40	17.43 ± 10.61	< 0.001^a^
YMRS, mean ± SD	0.13 ± 0.34	7.93 ± 7.51	< 0.001^a^
GAF, mean ± SD	90.47 ± 3.85	55.36 ± 11.46	< 0.001^a^
FAST, mean ± SD	5.38 ± 6.98	35.50 ± 16.79	< 0.001^a^
Illness Duration, mean ± SD	NA	11.61 ± 8.29	
Age at first mania episode, mean ± SD	NA	21.07 ± 8.03	
Age at first depressive episode, mean ± SD	NA	22.10 + 11.70	
Psychiatric medications (%)			
Lithium	NA	35.7%	
Antidepressants	NA	35.7%	
Atypical Antipsychotics	NA	42.9%	
Typical Antipsychotics	NA	0%	
Anticonvulsant	NA	42.9%	
Benzodiazepines	NA	28.6%	
Comorbidities (%)			
GAD	NA	14.3%	
PTSD	NA	28.6%	
Social Phobia	NA	21.4%	
PD	NA	28.6%	
Agoraphobia	NA	35.7%	

Abbreviations: GAD, Generalized Anxiety Disorder; FAST, Functional Assessment Screening Tool; GAF, Global Assessment of Functioning, MADRS, Montgomery-Asberg Depression Scale; NA, not applicable; PD, Personality disorders; PTSD, Post-traumatic stress disorder; YMRS, Young Mania Rating Scale.

a Kruskal–Wallis test

b chi-square test.

**Table 2:** Summary of univariate ANOVA on the enzymatic activities for mitochondrial respiratory chain (complexes I, II, and V), citrate synthase, and the mitochondrial genome (mtDNA) in peripheral blood mononuclear cells (PBMCs) from healthy controls (HCs) and patients with Disorder type I (BD-I).

Dependent Variables	Independent Variables	SS	*df*	MS	*F*	*Sig*.
**Complex I**	Corrected Model	2.513^a^	5	0.503	3.290	0.021
R^2^adj = 0.283	Intercept	0.922	1	0.922	6.037	0.022
	Age	0.056	1	0.056	0.367	0.550
	Sex	0.029	1	0.029	0.192	0.665
	BMI	0.001	1	0.001	0.006	0.937
	Smoking status	0.014	1	0.014	0.094	0.762
	HCs vs BD-I	1.949	1	1.949	12.757	**0.002**
**Complex II**	Corrected Model	4.645^a^	5	0.929	7.358	0.000
R^2^adj = 0.523	Intercept	0.134	1	0.134	1.058	0.314
	Age	0.430	1	0.430	3.404	0.077
	Sex	0.216	1	0.216	1.711	0.203
	BMI	0.156	1	0.156	1.233	0.278
	Smoking status	0.444	1	0.444	3.519	0.073
	HCs vs BD-I	4.078	1	4.078	32.299	**< 0.001**
**Complex IV**	Corrected Model	7.311	5	1.462	4.752	0.004
R^2^adj = 0.393	Intercept	0.434	1	0.434	1.412	0.246
	Age	0.500	1	0.500	1.624	0.215
	Sex	0.435	1	0.435	1.414	0.246
	BMI	0.036	1	0.036	0.116	0.736
	Smoking status	0.162	1	0.162	0.528	0.474
	HCs vs BD-I	5.290	1	5.290	17.191	< 0.001
**Citrate Synthase**	Corrected Model	409.328	5	81.866	1.694	0.174
R^2^adj = 0.107	Intercept	114.942	1	114.942	2.378	0.136
	Age	33.444	1	33.444	0.692	0.414
	Sex	108.902	1	108.902	2.253	0.146
	BMI	4.378	1	4.378	0.091	0.766
	Smoking status	9.119	1	9.119	0.189	0.668
	HCs vs BD-I	251.264	1	251.264	5.198	**0.032**
**mtDNAcn**	Corrected Model	4.804	5	0.961	1.611	0.195
R^2^adj = 0.095	Intercept	0.694	1	0.694	1.164	0.291
	Age	0.789	1	0.789	1.324	0.261
	Sex	0.476	1	0.476	0.798	0.381
	BMI	2.569	1	2.569	4.308	0.049
	Smoking status	0.030	1	0.030	0.051	0.824
	HCs vs BD-I	1.170	1	1.170	1.963	0.174

**Table 3:** Results from Multiple Linear Regression of Montgomery-Asberg Depression Scale (MADRS), Young Mania Rating Scale (YMRS), mitochondrial health index (MHI) and circulating cell-free mitochondrial DNA (ccf-mtDNA).

Dependent Variables	Independent Variables	*B*	*SE B*	*β*	*Sig*.
**MADRS**	(Constant)	42.217	19.038		0.057
R^2^adj = 0.705	Sex	1.562	3.678	0.076	0.682
p = 0.008	Age	0.018	0.209	0.015	0.935
	Smoking (no/yes)	4.494	4.036	0.199	0.298
	BMI	−0.063	0.287	−0.038	0.831
	MHI	−0.440	0.109	−0.798	**0.004**
**YMRS**	(Constant)	1.418	25.379		0.957
R^2^adj = −0.043	Sex	8.585	4.904	0.586	0.118
p = 0.529	Age	0.176	0.279	0.211	0.545
	Smoking (no/yes)	−4.720	5.381	−0.294	0.406
	BMI	−0.299	0.382	−0.251	0.456
	MHI	0.041	0.145	0.105	0.785
**MADRS**	(Constant)	−16.777	12.147		0.205
R^2^adj = 0.586	Sex	0.967	4.507	0.047	0.836
p = 0.027	Age	0.164	0.232	0.139	0.499
	Smoking (no/yes)	7.235	4.808	0.320	0.171
	BMI	−0.333	0.367	−0.197	0.392
	ccf-mtDNA	0.033	0.011	0.734	**0.016**
**YMRS**	(Constant)	7.011	13.704		0.623
R^2^adj = −0.048	Sex	8.543	5.084	0.584	0.131
p = 0.535	Age	0.158	0.262	0.189	0.564
	Smoking (no/yes)	−4.946	5.424	−0.308	0.388
	BMI	−0.282	0.414	−0.236	0.516
	ccf-mtDNA	−0.003	0.012	−0.080	0.840
**MHI**	(Constant)	167.669	28.489		< 0.001
R^2^adj = 0.605	Sex	−13.082	7.842	−0.336	0.139
p = 0.029	Age	−0.343	0.610	−0.138	0.592
	Smoking (no/yes)	−18.868	9.471	−0.426	0.087
	Length of illness	−1.949	0.649	−0.764	**0.020**
**ccf-mtDNA**	(Constant)	−378.33	335.176		0.296
R^2^adj = 0.666	Sex	212.173	92.264	0.426	0.055
p = 0.017	Age	1.181	7.181	0.037	0.874
	Smoking (no/yes)	223.772	111.423	0.395	0.085
	Length of illness	26.422	7.641	0.810	**0.011**
**GAF**	(Constant)	25.662	24.804		0.312
R^2^adj = 0.521	Sex	−12.019	5.703	−0.311	0.046
p = 0.003	Age	0.108	0.358	0.047	0.765
	Smoking (no/yes)	4.898	7.683	0.096	0.530
	BMI	0.691	0.517	0.212	0.194
	MHI	0.344	0.081	0.702	**< 0.001**
**FAST**	(Constant)	17.052	21.421		0.434
R^2^adj = 0.553	Sex	9.441	5.580	0.244	0.104
p = 0.002	Age	0.239	0.320	0.107	0.464
	Smoking (no/yes)	11.306	7.528	0.218	0.146
	BMI	−0.350	0.500	−0.106	0.491
	MHI	−0.301	0.063	−0.711	**< 0.001**
**GAF**	(Constant)	99.007	24.705		< 0.001
R^2^adj = 0.264	Sex	−8.718	6.754	−0.223	0.21
p = 0.035	Age	−0.255	0.390	−0.111	0.52
	Smoking (no/yes)	8.539	8.606	0.168	0.332
	BMI	0.221	0.617	0.063	0.724
	ccf-mtDNA	−0.042	0.014	−0.528	**0.005**
**FAST**	(Constant)	−45.281	23.291		0.064
R^2^adj = 0.324	Sex	3.704	6.414	0.095	0.569
p = 0.014	Age	0.378	0.355	0.169	0.299
	Smoking (no/yes)	7.782	8.218	0.151	0.353
	BMI	0.161	0.590	0.046	0.787
	ccf-mtDNA	0.046	0.012	0.590	**0.001**
